# Angiogenic Properties of Concentrated Growth Factors (CGFs): The Role of Soluble Factors and Cellular Components

**DOI:** 10.3390/pharmaceutics13050635

**Published:** 2021-04-29

**Authors:** Nadia Calabriso, Eleonora Stanca, Alessio Rochira, Fabrizio Damiano, Laura Giannotti, Benedetta Di Chiara Stanca, Marika Massaro, Egeria Scoditti, Christian Demitri, Paola Nitti, Andrea Palermo, Luisa Siculella, Maria Annunziata Carluccio

**Affiliations:** 1 National Research Council (CNR), Campus Ecotekne, Institute of Clinical Physiology (IFC), University of Salento, Via per Monteroni, 73100 Lecce, Italy; nadia.calabriso@ifc.cnr.it (N.C.); marika.massaro@ifc.cnr.it (M.M.); egeria.scoditti@ifc.cnr.it (E.S.); 2 Laboratory of Molecular Biology, Department of Biological and Environmental Sciences and Technologies, Campus Ecotekne, University of Salento, Via per Monteroni, 73100 Lecce, Italy; eleonora.stanca@unisalento.it (E.S.); alessio.rochira@unisalento.it (A.R.); fabrizio.damiano@unisalento.it (F.D.); laura.giannotti@unisalento.it (L.G.); benedetta.dichiara@unisalento.it (B.D.C.S.); 3 Department of Engineering for Innovation, Campus Ecotekne, University of Salento, Via per Monteroni, 73100 Lecce, Italy; christian.demitri@unisalento.it (C.D.); paola.nitti@unisalento.it (P.N.); 4 Implant Dentistry College of Medicine and Dentistry Birmingham, University of Birmingham, Birmingham B4 6BN, UK; andrea.palermo2004@libero.it

**Keywords:** angiogenesis, biomaterials, concentrated growth factors (CGFs), endothelial cells, endothelial markers, endothelial progenitor cells (EPCs), matrix metalloproteinases, pro-angiogenic factors, tissue regeneration, vasculogenesis

## Abstract

Blood-derived concentrated growth factors (CGFs) represent a novel autologous biomaterial with promising applications in regenerative medicine. Angiogenesis is a key factor in tissue regeneration, but the role played by CGFs in vessel formation is not clear. The purpose of this study was to characterize the angiogenic properties of CGFs by evaluating the effects of its soluble factors and cellular components on the neovascularization in an in vitro model of angiogenesis. CGF clots were cultured for 14 days in cell culture medium; after that, CGF-conditioned medium (CGF-CM) was collected, and soluble factors and cellular components were separated and characterized. CGF-soluble factors, such as growth factors (VEGF and TGF-β1) and matrix metalloproteinases (MMP-2 and -9), were assessed by ELISA. Angiogenic properties of CGF-soluble factors were analyzed by stimulating human cultured endothelial cells with increasing concentrations (1%, 5%, 10%, or 20%) of CGF-CM, and their effect on cell migration and tubule-like formation was assessed by wound healing and Matrigel assay, respectively. The expression of endothelial angiogenic mediators was determined using qRT-PCR and ELISA assays. CGF-derived cells were characterized by immunostaining, qRT-PCR and Matrigel assay. We found that CGF-CM, consisting of essential pro-angiogenic factors, such as VEGF, TGF-β1, MMP-9, and MMP-2, promoted endothelial cell migration; tubule structure formation; and endothelial expression of multiple angiogenic mediators, including growth factors, chemokines, and metalloproteinases. Moreover, we discovered that CGF-derived cells exhibited features such as endothelial progenitor cells, since they expressed the CD34 stem cell marker and endothelial markers and participated in the neo-angiogenic process. In conclusion, our results suggest that CGFs are able to promote endothelial angiogenesis through their soluble and cellular components and that CGFs can be used as a biomaterial for therapeutic vasculogenesis in the field of tissue regeneration.

## 1. Introduction

Over recent decades, numerous efforts have been made in the field of regenerative medicine; however, clinical applications of tissue engineering constructs are still scarce [1].

Major limitations in this field are related to the inadequate blood vessel network, which is crucial to ensure oxygen diffusion and nutrient supply and critical for the successful implantation of the tissue graft [2]. Therefore, strategies that promote vascularization by inducing angiogenesis and/or vasculogenesis may play an important role in tissue regeneration. Angiogenesis, the growth of new capillaries from pre-existing vessels by mature endothelial cells, and vasculogenesis, the de novo vessel formation by bone marrow derived endothelial progenitor cells (EPCs), play important roles in postnatal neo-vascularization [3,4]. According to the initial finding, EPCs were defined as cells positive for both hematopoietic stem cell marker CD34 or CD133 and for endothelial marker proteins, such as VEGFR-2 [5]. They can be mobilized from bone marrow into the blood circulation and thus colonize the vascularization sites and differentiate into mature endothelial cells [4,6]. The development of new blood vessels is a multifactorial process regulated by an interplay of various growth factors, such as vascular endothelial growth factor (VEGF), transforming growth factor-beta (TGF-β), platelet-derived growth factor (PDGF), basic fibroblast growth factor (FGF-2) and angiopoietins (Ang) [7,8,9,10,11,12,13]. VEGF is the master regulator of angiogenesis [9,10]. It regulates the angiogenesis of mature endothelial cells [10,14], enhances EPC proliferative and migratory activity, mediates the differentiation of EPCs into endothelial cells, and, together with CXC motif chemokine ligand-12 (CXCL-12, also called stromal cell derived factor-1, SDF-1), drives the homing of EPCs into the site of vascular injury [15]. VEGF stimulates endothelial cells to express and release matrix metalloproteinases (MMPs), which, by degrading the extracellular matrix, allow endothelial cells to migrate into interstitial space, where they form buds and capillary shoots [16,17].

Therapeutic angiogenesis is required both for rapid vascularization of tissue-engineered constructs and for the treatment of ischemic conditions. While ischemic tissues are already vascularized and proangiogenic therapy aims to expand the microvascular networks to promote collateral artery remodeling and restore physiological blood flow, tissue-engineered grafts are avascular upon implantation and need to attract vascular in-growth. In order to rapidly guide sprouting of new vessels and their migration toward the graft core, it is desirable that the graft matrix presents an optimized microenvironment of angiogenic cues. This can be achieved through several strategies, including the functionalization of biomaterials with pro-angiogenic factors, nucleic acids, and angiogenic cells, by predecorating a suitable material (such as fibrin or collagen) with optimized doses and combinations of angiogenic factors and/or by employing a decellularized extracellular matrix enriched in morphogens by suitable progenitor cell lines [18,19].

However, the exogenous application of angiogenic growth factors in tissue engineering does not always achieve success in vivo due to their rapid diffusion, short half-life time, and rapid proteolysis [20]. The failure of growth factor therapies has been also explained by their poor retention kinetics and the use of either insufficient or excessive doses. Growth factors, such as VEGF or FGF-2, presented intrinsically low stability with an active half-life following intravenous injection, by about 50 min or 3 min, respectively [20]. Moreover, direct injection of growth factors, such as BMP-2, in high enough doses in order to reach and sustain a sufficiently high local concentration to be effective may result in side effects in vivo.

Thus, proper delivery systems are essential to stabilize growth factors and provide long-term sustained and controlled release for in vivo efficacy.

Considering the multistep process of angiogenesis, systems to control the delivery of multiple angiogenic mediators in a spatiotemporal manner are necessary [10].

Various strategies have been performed for the controlled release of growth factors, including encapsulated VEGF in alginate hydrogel or conjugating VEGF into a collagen scaffold. Another approach was the pre-encapsulation of growth factors into microspheres, followed by the incorporation of the microspheres into scaffolds. The first system capable of providing multiple growth factors in a space-time manner, developed by Mooney et al. [21], attempted to mimic physiological angiogenesis by providing VEGF to stimulate blood vessel formation followed by PDGF delivery to ensure vessel maturation and stabilization. By loading VEGF directly into the scaffold and PDGF within the microspheres entrapped within the scaffold, a multi-modal delivery system was developed, allowing for the rapid release of VEGF and slower release of PDGF. The dual delivery of VEGF and PDGF produced an increase in vessel quantity and size in vivo, confirming the relevance for precise control over the spatiotemporal release of multiple factors [19].

However, as therapeutic vasculogenesis has failed to be translated clinically, there is a clear need for the design and development of new systems to allow for the translation of safe and efficacious treatments to induce angiogenesis.

Recent advances in regenerative medicine have increased the shift from heterologous to autologous therapies. In this context, various techniques have been developed to process peripheral blood in order to obtain useful hemoderivative products for wound healing and therapeutic angiogenesis [22].

Depending on their contents of platelets leucocytes and fibrin architecture, hemoderivative products are commonly classified as platelet rich plasma (PRP), platelet poor plasma (PPP), and platelet rich fibrin (PRF) [19]. Concentrated growth factors (CGFs), developed by Sacco in 2006, are the latest generation of platelet concentrate products [20]. The CGF is produced by the centrifugation of venous blood without the addition of any exogenous product and is therefore free from cross-contamination. Repeated switch of the centrifugation speed results in the production of CGFs as a modified form of PRF, with high amounts of cytokines, platelets, nucleated cells, and very dense fibrin scaffolds [23,24,25,26,27,28,29]. Fibrin binds to various growth factors, including VEGF, PDGF, FGF-2, IGF, and TGF-β1, thus acting as a scaffold to protect growth factors from proteolytic degradation and release them slowly for a longer time [30,31].

The CGF, as a fibrin scaffold, is considered a reservoir of natural growth factors, which can be released gradually over a period of time and play a crucial role in hard and soft tissue repair [32].

Several studies have investigated the effects of CGFs on tissue regeneration of alveolar and sinus bone, fracture repair, and implant stability, displaying a good tissue regenerative property [29,33,34,35]. Some research also showed the effects of CGFs on the osteogenic differentiation of rabbit periosteum-derived cells and human bone marrow stem cells in vitro [36,37], and reported CD34 positive stem cells in CGFs [24,37]. However, to the best of our knowledge, the CGF effects on the neo-vascularization process have not yet been fully characterized.

In this study, we aimed to determine the role of CGFs in neo-angiogenesis, evaluating the contribution of soluble factors and CGF-released cells. To this aim, we cultured CGFs for appropriate time periods to pursue the highest content of released growth factors. The effects of CGF-soluble factors on the migration and tubular structure formation of human endothelial cells, as well as on endothelial expression of pro-angiogenic factors, were evaluated. At the same time, CGF-released cells were characterized by assessing the expression of stem cells and mature endothelial cells markers, and their contribution to angiogenesis was explored.

## 2. Materials and Methods

### 2.1. Materials

The cell culture medium, gelatin, fetal bovine serum, glutamine, penicillin and streptomycin were obtained from Merck (Merck Life Science S.r.l., Milan, Italy). Human enzyme-linked immunosorbent assay (ELISA) kits were obtained from Cusabio (Cusabio Biotech, Wuhan, China) for determination of VEGF and TGF-β1 and from R&D (R&D Systems Inc, Minneapolis, MN, USA) for determination of BMP-2 and metalloproteinases MMP-2 and MMP-9. Primary antibodies against CD34, eNOS, and VE-cadherin were purchased from Santa Cruz Biotechnology, as well as biotinylated anti-mouse IgG and biotinylated anti-rabbit IgG antibodies. The primary antibody against VEGFR-2 was purchased from Cell Signaling (Cell Signaling Technology, Milan, Italy). Extravidin peroxidase and diamminobenzidine were obtained from Merck, as well all other reagents, unless otherwise indicated.

### 2.2. Preparation of CGFs and CGF-Conditioned Medium

**Figure 1:**
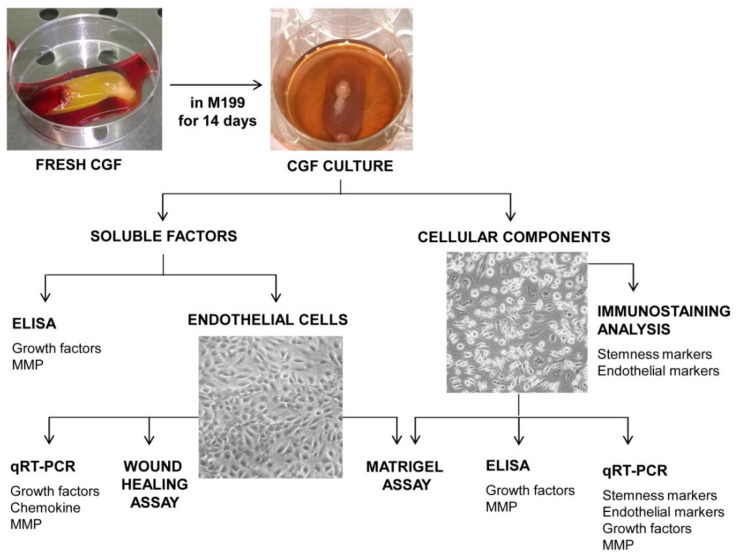
Experimental design.

Venous blood (8 mL) from 5 healthy, non-smoking adult donors, two females and three males, aged between 27 and 50 years old, was collected and immediately centrifuged by a Medifuge device (Medifuge MF200; Silfradent srl, Forlì, Italy), at 25 °C, using a program with the following characteristics: 30 s acceleration, 2 min 2700 rpm, 4 min 2400 rpm, 4 min 2700 rpm, 3 min 3000 rpm, and 36 s deceleration and stop, to obtain CGF clot, as previously described [36,38]. Informed consent was obtained from the donors included in this study in accordance with Declaration of Helsinki. For each set of experiments, CGFs were prepared from the same blood sample of a single donor. To remove excess serum, CGF clot was washed with phosphate-buffered saline (PBS) and cultured in sterile dish with 2 mL of M199 medium at 37 °C in a humidified atmosphere with 5% CO_2_ for 14 days. Then, CGF clot was removed, and CGF-conditioned medium (CGF-CM) was collected and centrifuged at 1500 rpm for 10 min. Then, the supernatant was stored at −80 °C until use, and the pellet was used for the CGF cell culture. In particular, CGF-CM was analyzed to evaluate the content of growth factors and metalloproteinases and its effects on the angiogenesis of human endothelial cells (Figure 1).

### 2.3. CGF Cell Culture and Characterization

CGF-released cells were suspended in M199 medium complete, containing 10% FBS, 2 mM glutamine, 100 U/mL penicillin, and 100 μg/mL streptomycin and seeded in appropriate cell plate coated with 1% gelatin. In our experimental conditions, CGF-derived cells adhered to the culture plate and grew until they reached the confluence. CGF-derived cells were phenotypically characterized by hematoxylin–eosin staining and immunostaining analysis (Figure 1). CGF cells, grown in 24-well plates on coverslips (Thermanox, ProSciTech, Thuringowa, Queensland, Australia), were fixed with cold methanol for 10 min on ice and washed with PBS; then, hematoxylin–eosin staining and immunostaining analysis were performed. For immunocytochemistry, monolayers were incubated overnight with antibodies against endothelial nitric oxide synthase (eNOS), VE-cadherin, and CD34 at concentrations of 1 μg/mL and with the antibody against VEGFR-2 at a concentration of 0.2 μg/mL. After 3 washes with PBS, monolayers were incubated for 1 h with a biotinylated anti-mouse (for eNOS, VE-cadherin) or anti-rabbit (for VEGFR-2, CD34) IgG antibody (Santa Cruz), and for 1 h with extravidin peroxidase (Merck). Monolayers were then incubated with diamminobenzidine (Merck) for 30 min, washed, and directly mounted, and pictures (×100 magnification) were taken with a digital output Canon Powershot S50 camera.

### 2.4. Endothelial Cell Culture and Treatment

Human umbilical vein endothelial cells (HUVEC) were obtained from discarded umbilical cords and treated anonymously, conforming to the principles outlined in the Declaration of Helsinki, after specific permission was granted by the local health authority. HUVECs were grown on cell plates, pre-coated with 1% gelatin, in M199 medium containing 10% fetal bovine serum (FBS), 100 U/mL penicillin, and 100 μg/mL streptomycin, as described in [39], and utilized up to the fifth passage from primary cultures. The human microvascular endothelial cell line (HMEC-1), obtained from Dr. Thomas J. Lawley, was cultured as previously described [40]. All experiments were performed in HUVECs and in HMEC-1. For treatment, confluent endothelial cells were shifted to the medium, supplemented with 3% FBS, and subsequently treated with increasing concentrations of CGF-CM (1%, 5%, 10% and 20%) for 16 h. CGF-CM concentrations were calculated on the basis of the volume of CGF-CM that was added to the total volume of the culture medium.

### 2.5. Cell Migration and Tube Formation Assays

To evaluate cell migration, endothelial cells were seeded into 6-well plates at the density of 2 × 10^5^ cells/well until confluence, and then a scratch wound was performed with a sterile microtip. After washing with PBS to remove detached cells, the first series of photos were taken with an attached digital output Canon Powershot S50 camera (0 h). Monolayers were incubated with increasing concentrations of CGF-CM (1%, 5%, 10%, and 20%) or 3% FBS (control) for 16 h and then washed and again photographed (16 h). Cell repair of the wound was determined by measuring the width (μm) of the denuded area along the scratch (at five different levels) using the Optimas Image analysis software (Media Cybernetics, Pleasanton, CA, USA).

The formation of vascular-like structures by endothelial cells was assessed on the growth factor-reduced basement membrane matrix “Matrigel” (11.1 mg/mL; Becton Dickinson Biosciences, Bedford, MA, USA), as previously described [40,41]. The bottoms of 24-well culture plates were coated with Matrigel (50 μL per well) diluted at 1:2 with M199 medium. After gelatinization at 37 °C for 30 min, gels were overlaid with 500 μL of 3% FBS-containing M199 medium containing 4 × 10^4^ cells per well in the presence of CGF-CM (1%, 5%, 10%, and 20%) or 3% FBS (control) and then incubated for 16 h at 37 °C.

Tube formation was monitored by inverted phase-contrast microscopy (Leica, Wetzlar, Germany), and pictures (×100 magnification) were taken with an attached digital output Canon Powershot S50 camera. Tube formation was quantified by counting the tubule branching points in three randomly selected fields per well and was expressed as branch points per field. To evaluate the contribute of CGF-derived cells in tube-like formation, 1 × 10^4^ CGF-derived cells were loaded with DilC18 vital fluorescent dye and seeded on Matrigel together with mature endothelial cells (3 × 10^4^). After 16 h, tube formation was monitored by light microscopy and fluorescence microscopy. Fluorescence images were captured by NIS-Elements F 3.0.

### 2.6. Growth Factors and Metalloproteinases Release

CGF-CM as well as the supernatants of CGF-derived cells and CGF-CM-treated endothelial cells were collected, and the growth factors VEGF and TGF-β1 and the metalloproteinases MMP-9 and MMP-2 were quantified using commercial human ELISA kits, according to the manufacturer’s instructions. To detect the total amount of TGF-β1, the latent form of TGF-β1 was first converted into the active form according to the manufacturer’s instructions. Specifically, endothelial cells were incubated with CGF-CM (10%) for 16 h, and then the culture media were replaced with fresh medium. After 48 h, supernatants were collected and analyzed. CGF-derived cells were grown until confluence in M199 complete; then, the culture media were replaced with fresh medium and incubated for 48 h; later, supernatants were collected and analyzed.

All results are expressed as ng of angiogenic factors per mL volume of supernatants/conditioned medium.

### 2.7. Quantitative Reverse Transcription–Polymerase Chain Reaction Analysis

Total RNA was isolated from CGF-derived cells and CGF-CM-treated endothelial cells using the TRIzol reagent (Invitrogen) according to the manufacturer’s protocol. For quantitative polymerase chain reaction, total RNA (1 μg) was converted into first-strand cDNA using the High-Capacity cDNA Reverse Transcription Kit (Applied Biosystems, Monza, Italy). The quantitative RT-PCR was performed in the Bio-Rad Biosystems CFX384 Touch Real-Time PCR Detection System using SYBR Green PCR Master Mix. The human cDNA fragments were amplified using primers synthesized by Thermo Fisher (Thermo Fisher Scientific, Rodano, Italy) and are reported in Table 1. We explored the expression of the following genes: VEGF, TGF-β1, BMP-2, MMP-9, MMP-2, Ang-1, Ang-2, PDGF-B, FGF-2, VE-cadherin, eNOS, VEGFR-2, CD31, CD133, CD34, CXCL-12, and CXCR-4. The quantifications were performed using the efficiency-adjusted ΔΔCT method (CFX Manager), with GAPDH as an internal control.

### 2.8. Statistical Analysis

Values are expressed as mean ± SD for the number of experiments indicated in the legends of the figures. Differences between two groups were determined by unpaired Student’s *t*-test. Multiple comparisons were performed by one-way analysis of variance (ANOVA), and individual differences were then tested by Fisher’s protected least significant difference test after the demonstration of significant intergroup differences by ANOVA. Differences between means from at least three independent experiments with *p* < 0 05 were considered statistically significant.

## 3. Results

In an attempt to mimic the natural release of factors by CGFs that could occur in vivo, we cultured CGF in M199 medium for an appropriate time. According to previous studies showing 14 days as the best time to obtain the highest growth factor content [42,43], we chose this time for the CGF culture. After that, we collected the CGF-conditioned medium (CGF-CM) and used it to quantify angiogenic factors and to analyze the angiogenic response of human endothelial cells. At the same time, CGF-derived cells, adhering to plates and able to grow and propagate, were phenotypically characterized, and their contribution to angiogenesis was evaluated (Figure 1).

### 3.1. CGF-Soluble Components Improved Endothelial Angiogenesis

In order to analyze the pro-angiogenic effects of soluble factors released by CGFs in culture medium, we analyzed the CGF-CM content of angiogenic factors by ELISA. As reported in Table 2, during the culture period, CGF released two main growth factors, VEGF and TGF-β1. Indeed, in CGF-CM, VEGF and TGF-β1 reached concentrations of about 1.1 ± 0.1 and 18.3 ± 1.2 ng/mL, respectively. Another mediator of the TGF family, BMP-2, was also released by CGFs. Moreover, CGF-CM was enriched with matrix metalloproteinases MMP-2 and MMP-9, and their concentrations were 136.6 ± 25.5 and 490.7 ± 59.7 ng/mL, respectively (Table 2).

**Figure 2:**
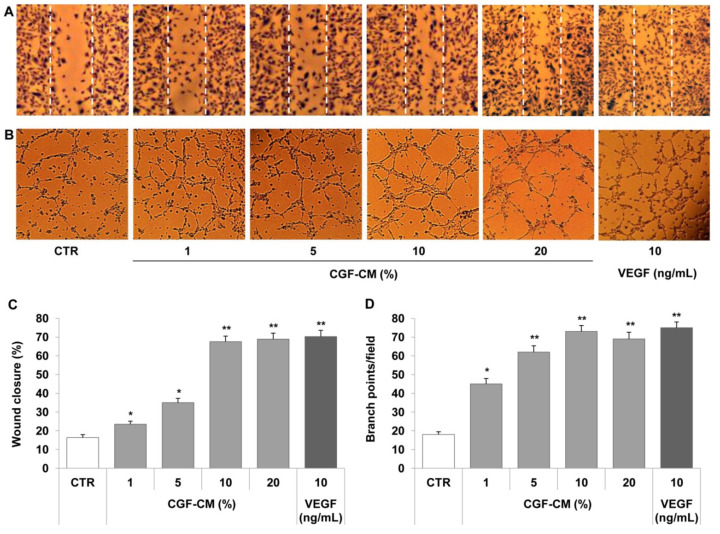
CGFs promote endothelial cell migration and tube formation. A scratch wound was performed on endothelial monolayers of HMEC-1 that were stimulated with CGF-CM (1%, 5%, 10% or 20%) for 16 h (**A**). Cell migration was quantified and monitored under phase-contrast microscopy (**C**). HMEC-1 cells were plated onto a three-dimensional collagen gel (Matrigel) surface and then stimulated with CGF-CM (1%, 5%, 10%, or 20%) or VEGF (10 ng/mL) for 16 h (**B**,**D**). Tube formation was monitored under phase-contrast microscopy, photographed, and analyzed. Images are representative of cell migration (**A**) and capillary-like tube formation (×100 magnification) (**B**). Data are representative of three independent experiments, expressed as means ± SD, and presented as percentage of wound closure (**C**) and branch points per field (**D**). Each experiment consisted of four replicates for each condition. * *p* < 0.05 and ** *p* < 0.01 vs. control (CTR).

Since CGFs released factors boosting the angiogenic process, we determined the effects of CGF-CM on the endothelial angiogenic response, evaluating cell migration and tube-like structure formation in human endothelial cells. For this purpose, endothelial cells, HUVECs or HMEC-1, were treated with CGF-CM at different concentrations (1%, 5%, 10%, or 20%) for 16 h. The representative scratch wound healing images (Figure 2A) and the related bar graph (Figure 2C) show that CGF-CM induced the migration of HMEC-1. Moreover, CGF-CM augmented endothelial angiogenic activity by increasing the capillary-like tube formation, as indicated by the increased number of branch points on Matrigel (Figure 2B,D). Similar results were obtained in HUVEC. Overall, CGF-CM sustained cell migration and the angiogenic ability of endothelial cells in a concentration-dependent manner, with a maximum significant effect at the concentration of 10% (Figure 2), which was the dose chosen for the subsequent characterization of molecular mechanisms. The effect of 10% CGF on the promotion of angiogenesis was similar to that achieved by stimulating endothelial cells with a high concentration of VEGF (10 ng/mL) (Figure 2).

### 3.2. CGF-Soluble Components Induced Pro-Angiogenic Factor Expression in Endothelial Cells

**Figure 3:**
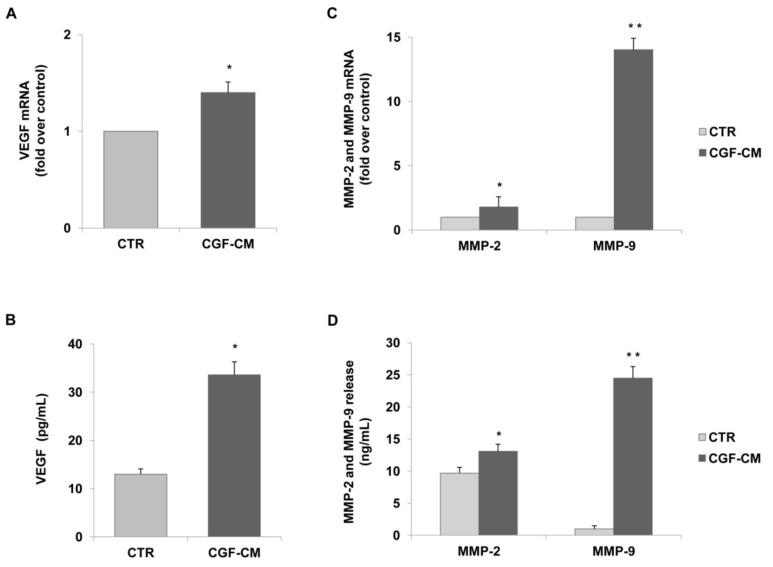
CGFs induce the expression and release of VEGF and matrix metalloproteinases in endothelial cells. HUVECs were incubated with CGF-CM (10%) or culture medium (CTR) for 4 h and mRNA levels of VEGF. (**A**) MMP-9 and MMP-2 (**C**) were analyzed by qRT-PCR and expressed as fold over unstimulated control (CTR) (mean ± SD). HUVECs were incubated with CGF-CM (10%) or culture medium (CTR) for 4 h, and then culture media were removed and replaced with fresh media for 48 h. Media were collected, and VEGF (**B**), MMP-9, and MMP-2 (**D**) concentrations were analyzed by ELISA. Data are representative of three independent experiments, expressed as means ± SD. Each experiment consisted of four replicates for each condition. * *p* < 0.05 and ** *p* < 0.01 vs. control (CTR).

In order to explore the mechanisms underlying the CGF action on endothelial angiogenesis, we analyzed the effects of CGF-CM on the expression of the pro-angiogenic factor in endothelial cells. In particular, we analyzed the mRNA levels and protein release of VEGF, MMP-2, and MMP-9 by qRT-PCR and ELISA, respectively. The expression and release of VEGF in CGF-CM-treated endothelial cells were significantly up-regulated compared to those of untreated control cells (Figure 3A,B). In endothelial cells treated with 10% CGF-CM, the VEGF mRNA level was increased by about 1.4-fold compared to that of the control, and VEGF protein release reached values of 33.6 ± 2.7 pg/mL, while in control cells, this was 13.0 ± 1.1 pg/mL (Figure 3A,B). Moreover, CGF-CM also induced the mRNA expression of MMP-9 and, to a lesser extent, of MMP-2, which increased by about 14.0- and 1.8-fold compared to that of control, respectively (Figure 3C). According to the gene expression data, CGF-CM significantly induced the release of MMP-9 and MMP-2 in endothelial cells, reaching levels of 24.5 ± 1.8 and 13.5 ± 1.3 ng/mL, respectively, compared to that in controls (1.0 ± 0.2 for MMP-9 and 9.7 ± 0.8 for MMP-2) (Figure 3D).

**Figure 4:**
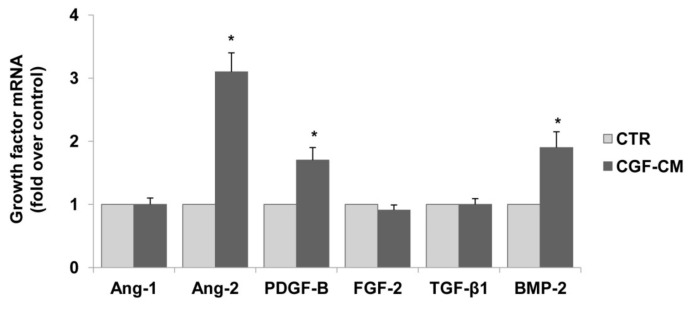
CGFs enhance the expression of pro-angiogenic factors in endothelial cells. HUVECs were incubated with CGF-CM (10%) or culture medium (CTR) for 4 hm and mRNA levels of Ang-1, Ang-2, PDGF-B, FGF-2, TGF-β1, and BMP-2 were analyzed by qRT-PCR and expressed as fold over unstimulated control (CTR) (mean ± SD). Each experiment consisted of four replicates for each condition. * *p* < 0.05 vs. CTR.

To enhance the pro-angiogenic mechanisms of CGFs, we analyzed the effect of CGF-CM on the endothelial expression of other pro-angiogenic factors, including Ang-1, Ang-2, and PDGF-B. As shown in Figure 4, CGF-CM up-regulated the mRNA levels of Ang-2, PDGF-B, and BMP-2 by about 3.1-, 1.7-, and 2.0-fold as compared to the control, but it left Ang-1, FGF-2, TGF-β1, mRNA levels unaffected.

### 3.3. Characterization and Angiogenic Properties of CGF-Derived Cells

**Figure 5:**
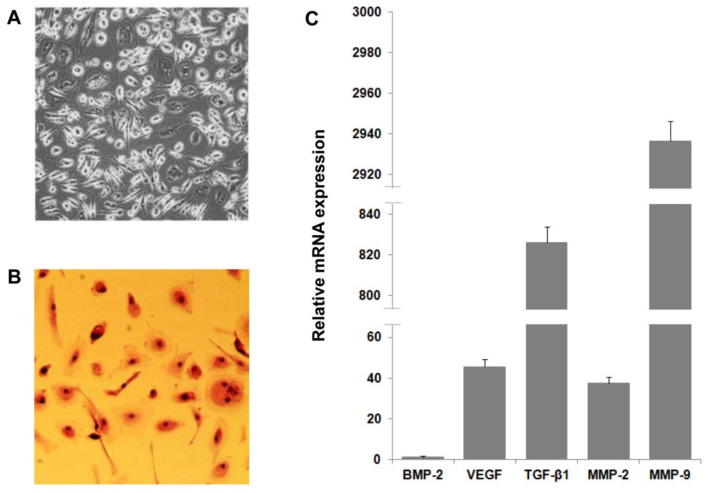
CGF-derived cell characterization and expression of pro-angiogenic factors. Cells were released by CGF clot and cultured in M199 medium (**A**). CGF cell morphological characterization was determined by hematoxylin–eosin staining (**B**). RNA was extracted from CGF-derived cells, and levels of pro-angiogenic factor expression were analyzed by qRT-PCR. GAPDH was considered a housekeeping gene. The expression level of genes is referred to with BMP-2 (=1) as the lesser expressed gene (**C**). Data are representative of three independent experiments.

In addition to providing soluble components, CGF also released numerous and heterogeneous cells, some of which were able to adhere to cell culture plates and to propagate (Figure 5A). We performed hematoxylin–eosin staining to analyze the morphology of these cells. Figure 5 shows CGF heterogeneous cells with spindle-shaped or round-like morphology. Evident nuclear staining was observed in all CGF-derived cells (Figure 5B).

To explore the role of these cells in the angiogenic potential of CGF, we analyzed the release of pro-angiogenic factors. As shown in Table 3, CGF-derived cells released VEGF and TGF-β1, which reached values of 0.021 ± 0.007 and 1.5 ± 0.4 ng/mL, respectively. Furthermore, these cells produced matrix metalloproteinases and released MMP-9 and MMP-2 into the medium at concentrations of 109.6 ± 13.8 and 16.7 ± 3.7 ng/mL, respectively (Table 3). The expression pattern of pro-angiogenic factors in CGF-derived cells was confirmed by qRT-PCR analysis (Figure 5C).

**Figure 6:**
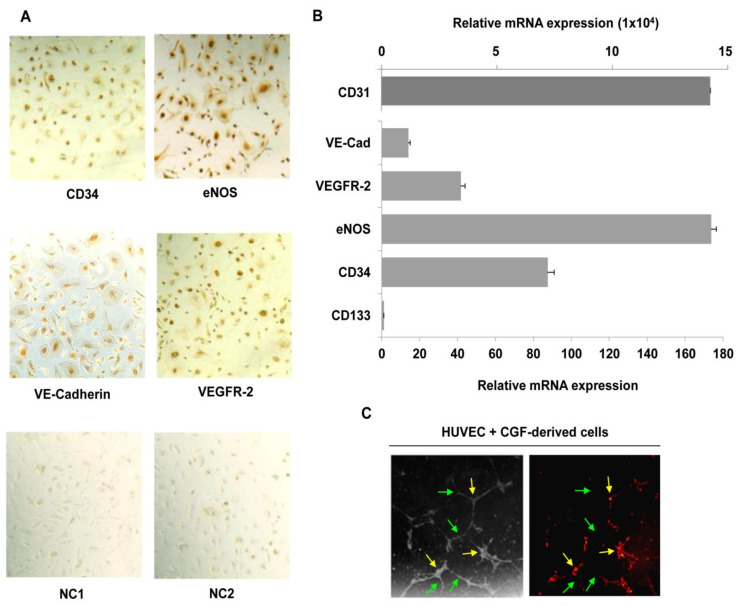
CGF-derived cells express hematopoietic stem cells and endothelial markers and participate in endothelial angiogenesis. Immunostaining analysis was performed for CD34, VE-cadherin, VEGFR-2 and eNOS (**A**), and negative controls for anti-mouse (NC1), and anti-rabbit (NC2) IgG antibodies were reported. RNA was extracted from CGF-derived cells, and the expression of hematopoietic stem cell (CD133 and CD34) and endothelial cell (CD31, VE-cadherin, VEGFR-2 and eNOS) markers was analyzed by qRT-PCR. GAPDH was considered a housekeeping gene. The expression level of genes is referred with CD133 (=1) as the lesser expressed gene (**B**). CGF-derived cells were loaded with DilC18 and plated with endothelial cells onto a Matrigel surface for 16 h. Tube formation was monitored and photographed. Images are representative of capillary-like tube formation under light microscopy on the left and fluorescence microscopy on the right (×100 magnification) (**C**). Green and yellow arrows indicate HUVEC and CGF-derived cells, respectively (**C**). Each experiment consisted of four replicates for each condition.

To further characterize CGF-derived cells, we investigated the expression of stem cell and endothelial markers. Using immunocytochemistry, we found that CGF-derived cells were positive for the hematopoietic stem cell marker CD34 and for endothelial markers eNOS, VEGFR-2, and VE-cadherin (Figure 6A). These findings suggest that CGF-derived cells are EPC-like cells. With qRT-PCR, we confirmed the positive expression for CD34, as well as that for CD31, eNOS, VEGFR-2, and VE-cadherin (Figure 6B). Moreover, among the tested genes, we reported that the endothelial marker CD31 exhibited the highest level of gene expression at the mRNA level, and CD133, the immature hematopoietic stem cell marker, the lowest level (Figure 6B). Interestingly, we found that CGF-derived cells, fluorescent dye labelled, functionally integrate into the endothelial capillary-like structures of mature endothelial cells (Figure 6C), contributing to angiogenic response.

**Figure 7:**
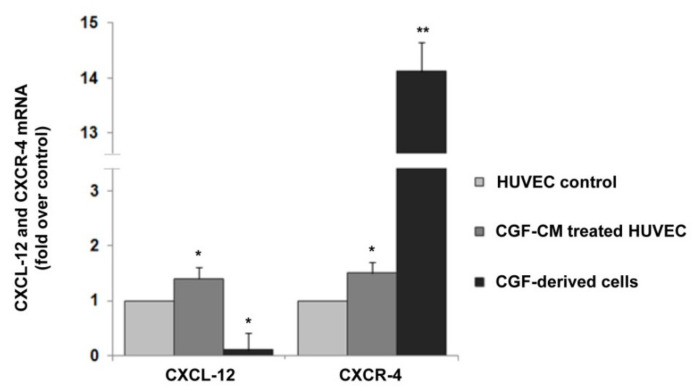
CXCL-12 and CXCR-4 expression in CGF-derived cells and CGF-CM-treated endothelial cells. mRNA was extracted from CGF-derived cells and CGF-CM (10%)-treated HUVECs for 4 h, and the expression levels of CXCL-12 and CXCR-4 were analyzed by qRT-PCR and expressed as fold over unstimulated HUVEC control (mean ± SD). Each experiment consisted of four replicates for each condition. * *p* < 0.05 and ** *p* < 0.01 vs. HUVEC control.

Finally, in EPC-like CGF-derived cells as well as in mature endothelial cells, we investigated the expression of CXCL-12 and CXCR-4, key regulators of the angiogenic process [44,45,46]. We found that CGF-CM significantly induced the expression of CXCL-12 and CXCR-4 in mature endothelial cells, whose expression was increased by about 1.4- and 1.5-fold compared to that of control, respectively. (Figure 7). Moreover, in CGF-derived cells, a strong CXCR-4 expression was observed, and its mRNA levels were higher by about 14.1-fold than those that in mature endothelial control cells (Figure 7). In contrast, the expression of CXCL-12 in CGF-derived cells was very low compared to that in endothelial control cells.

## 4. Discussion

In the present study, we reported novel properties of CGF in promoting neo-angiogenesis by releasing multiple soluble angiogenic factors and EPC-like cells.

It is well known that the formation of new blood vessels, crucial in tissue growth and repair, occurs through a complex process that requires the cooperation of several angiogenic factors responsible for multiple events, such as the budding of pre-existing resident endothelial cells and the recruitment of bone marrow derived EPC [3].

Currently, platelet concentrates are widely used to regenerate damaged tissues in the field of orthopedic and oral surgery [47,48,49,50,51]. CGFs, the latest generation of platelet concentrate products, are particularly enriched in growth factors due to a specific process that guarantees the intrinsic coagulation reaction in the venous blood causing the activation of CGFs [24]. Moreover, the dense fibrin network of CGFs leads to the protection of growth factors and chemokines as well as potentially a better sustained release [28]. Previous studies showed two phases in the release of growth factors by CGFs [52]: an immediate phase, which could be attributed to the instantaneous release from activated platelets during centrifugation or to simple diffusion; a late phase with accumulation peaks at 14 days, which could be explained by the release of growth factors after degradation of the fibrin structure and by the production of growth factors by the cells present in the CGFs [42,53,54,55]. However, there are different results in the literature concerning the release kinetics of growth factors, which could be due to different volumes of blood samples, observation times, and methods of extracting growth factors [54,55]. In many studies, CGFs have been manipulated using freeze–thaw and lyophilizing methods to extract and quantify growth factors [27,56]. Unlike these studies, we did not manipulate the CGFs after their preparation, but in order to mimic the natural release of soluble factors that could occur from the application of CGF in vivo, we cultured the CGFs in a culture medium for two weeks. We then collected the CGF-conditioned medium and analyzed the content of specific angiogenic factors and their effect on endothelial angiogenesis. Our data show that CGFs were able to release growth factors, including VEGF and TGF-β1, which reached amounts in the culture medium comparable to those recorded by Borsani et al. [57], but observed at longer times (14 days), according to other studies [42,54,55,58]. It was also reported that CGFs contained different angiogenesis-related growth factors, including PDGF, FGF-2, insulin-like growth factor-1 (IGF-1), and epidermal growth factor (EGF), which are of crucial importance in tissue regeneration [57,59,60].

In addition to growth factors, we found, for the first time, that CGFs released large amount of matrix metalloproteinases, such as MMP-2 and MMP-9, which play an essential role in angiogenesis. MMPs are extracellular endopeptidases that selectively degrade components of the extracellular matrix and allow endothelial cells to migrate and invade into the surrounding tissue and contribute to new vessel formation. Moreover, MMP-9 is involved in mobilizing EPCs and other progenitor cells from the bone marrow niche [16]. MMPs also liberate growth factors, including VEGF and TGF-β1, from the matrix-bound form [54]. These results improve our knowledge of CGF-soluble factors related to angiogenesis and are in agreement with previous studies showing the presence of MMPs in other platelet concentrate PRPs [61,62,63,64].

In order to evaluate the functional properties of CGF-released factors, we analyzed the effects of CGF-CM on the angiogenic response of mature endothelial cells, HUVECs and HMEC-1. We found that CGF-CM promoted the angiogenic potential of endothelial cell stimulation in a concentration dependent manner, cell migration, and tubule-like structure formation. The angiogenic response of endothelial cells induced by CGF can be, at least in part, explained by the presence of pro-angiogenic growth factors, such as VEGF and TGF-β1, which promote cell proliferation, migration, and differentiation in endothelial capillary structures.

MMPs, degrading extracellular matrix, also contribute to cell migration and vessel development. The endothelial angiogenic response was maximal when the concentrations of CGF-enriched medium reached 10%; higher concentrations did not guarantee stronger effects, as reported in other previous studies [34,42,65]. A rational explanation for this observation might be that CGFs contain not only pro-angiogenic factors that favor vascularization but also anti-angiogenic cytokines [65,66,67]. Here, we reported that the effect of 10% CGF-CM in promoting angiogenesis was similar to that achieved by stimulating endothelial cells with much higher concentration of VEGF, suggesting a synergistic action between VEGF and other pro-angiogenic factors in CGF-CM. Previous studies found that CGFs also contained FGF-2, an essential angiogenic growth factor, which could operate in coordination with other CGF-soluble factors in angiogenic process regulation [57,59,60]. Our findings show the angiogenic effect of CGFs as a complex of multiple mediators; however, the specific contribution of different CGF angiogenic factors requires further analysis through a loss-of-function approach, inactivating the different protein mediators using monoclonal antibodies or specific inhibitors blocking activated angiogenic pathways.

Soluble factors exerted an optimal effect on angiogenesis at low concentrations, which could correspond to those obtained in application of CGFs as a fibrin scaffold in vivo. Our results confirm and expand on a previous study reporting that CGFs, as extract, were able to induce endothelial cell proliferation, migration, and tubular structure formation [65]. Other studies have demonstrated the ability of CGFs to promote proliferation, migration, and angiogenesis of the dental pulp [34], as well as the proliferation, migration, and differentiation of human stem cells from the apical papilla [59].

Here, we discovered that CGF-CM significantly increased the expression and release of VEGF, MMP-9, and MMP-2 in cultured endothelial cells. Furthermore, CGF-CM up-regulated the expression of Ang-2, BMP-2, and PDGF-B, thus amplifying pro-angiogenic signals.

It is known that platelet concentrates could provide a supportive matrix for circulating stem/progenitor cells [68,69], which are recruited from blood to injured tissue by a variety of signaling molecules [5,70], including VEGF, MMP, PDGF-B, and Ang-2 [16,71,72,73]. Growing evidence points to the role of circulating CD34 positive cells, such as EPCs, in vascular maintenance, neovascularization, and angiogenesis [3,4,5,6,74]. Rodella et al. found CD34 positive cells in CGF [24]. The presence of CD34 positive cells that were dispersedly distributed in the fibrin network of a CGF membrane was also reported by Zhang et al. [37]. According to these studies, for the first time, we provide evidence on the ability of CGF culture to release CD34 positive cells capable of adhering to the substrate and growing for a relatively long time from CGF preparation. In addition to the expression of the CD34 hematopoietic stem cell marker, these cells also showed positive expression of endothelial markers, including that of eNOS, VE-cadherin, VEGFR-2, and CD31, suggesting that CGF-derived cells are EPCs. This result was confirmed by the ability of CGF-derived cells to take part in angiogenesis by incorporating themselves into tubule-like structures of mature endothelial cells. EPCs, normally present in bloodstream, could be trapped in the fibrin scaffold during CGF preparation, where the microenvironment rich in growth factors and cytokines could assure the survival and growth of EPCs. In accordance with the aptitude of EPCs to produce multiple angiogenic molecules, we also found that CGF-derived cells expressed and released growth factors VEGF and TGF-β1 and matrix metalloproteinases MMP-2 and MMP-9. This evidence could contribute to explaining the sustained quantity of pro-angiogenic factors released by CGFs. As reported for EPCs [45,46], we also found that CGF-derived cells expressed high levels of CXCR-4, which can be relevant to homing CGF cells to injured or ischemic sites. Moreover, CGF-CM significantly induced the expression of CXCL-12 and CXCR-4 in mature endothelial cells. CXCL-12 is a chemotactic molecule that promotes downstream effects mainly via the CXC motif chemokine receptor (CXCR)-4. It has been recognized that CXCL-12 was a strong chemoattractant for CD34 positive cells, including hematopoietic stem cells and EPC, which highly expressed CXCR-4, and induced their mobilization from bone marrow [75,76]. In addition, the increased expression of CXCL-12 in ischemic tissues carried out as a chemoattractant to support the homing of CXCR-4 positive EPC. Locally administered CXCL-12 also promoted EPCs accumulation at the site of ischemia, associated with ischemic neovascularization [77,78].

Our findings showing the ability of CGF to deliver CXCR-4 positive cells and to induce CXCL-12/CXCR-4 expression in mature endothelial cells reveal an additional mechanism in CGF-induced angiogenesis, which may have relevance in the neovascularization at the damage/ischemic site.

To date, there are no literature data regarding the implications of EPC-like cells present in CGF in clinical applications; thus, future studies should focus on this topic.

In our experimental conditions, based on the CGF culture, without any manipulation and preservation of its microenvironment, we have shown that CGFs are able to promote the process of neo-angiogenesis due to the synergistic action of multiple angiogenic mediators and EPCs, which, together with mature endothelial cells, could contribute to the vascularization at the site of CGF application.

In a previous study, we showed that VEGF was released from titanium dental implant surfaces permeated with CGFs and containing fibrin, which is fundamental to accommodate the cellular network [38]. The present results show that CGFs with their soluble factors, including VEGF, and their cellular components, such as EPC-like cells, promoted neo-angiogenesis, suggesting that the incorporation of CGFs on dental implant surfaces could produce a biologically active area capable of promoting intercellular communication and neo-angiogenesis during bone regeneration and healing.

Some limitations are involved in the present study. We evaluated the angiogenic properties of the CGF-conditioned medium, which may be related to the synergy of multiple mediators. Notwithstanding, the role of the specific angiogenic factor and its mechanism of action needs further investigation. In this study, we determined the angiogenic properties of non-manipulated CGFs, dissecting the role of soluble factors and, for the first time, also highlighting the contribution of EPC-like released cells. However, more studies are needed to further our understanding of the characteristics of CGF-released stem/progenitor cells and their role in the neo-angiogenesis. Moreover, our study was conducted in a preclinical model represented by HUVEC and HMEC-1 cultures, known as a reliable and versatile tool for studying in vitro angiogenesis. Despite this, the in vitro experimental results do not necessarily translate directly into the in vivo situation. Therefore, further investigations, including human trials, are needed to confirm and further evaluate the in vivo efficacy of CGFs in promoting vasculogenesis and tissue regeneration.

## 5. Conclusions

In the present study, our results suggest that CGFs represent not only a reservoir of multiple pro-angiogenic factors able to induce a pro-angiogenic phenotype in mature endothelial cells, but also a cell niche for EPC-like cells. Our in vitro model provides the first evidence that CGFs release EPC-like cells, which contribute to neo-angiogenesis producing pro-angiogenic factors and taking part in the formation of endothelial tubular structures.

CGFs function as a complex milieu that regulates, by means of a dense fibrin scaffold and multiple bioactive factors, the survival, growth, differentiation, and migration of immature cells, which could be a valid strategy to facilitate healing processes in tissue regeneration.

In conclusion, CGFs, with a direct effect on neo-angiogenic response, could represent a very promising biomaterial for therapeutic vasculogenesis and tissue healing, offering new and interesting perspectives for the use of CGFs in regenerative medicine.

However, our study was performed using an in vitro system, and therefore caution is required in translating these research findings in vivo.

## Figures and Tables

**Table 1:** Oligonucleotides used for quantitative real-time PCR analysis.

Gene Name	Accession Number	Forward Primer	Reverse Primer	Size (bp)
VEGF	NM_001171626.1	5′-GACACACCCACCCACATACA-3′	5′-TCTCCTCCTCTTCCCTGTCA-3′	215
TGF-β1	NM_000660.7	5′-CACGTGGAGCTGTACCAGAA-3′	5′-GAACCCGTTGATGTCCACTT-3′	219
BMP-2	NM_001200.4	5′-AGACCTGTATCGCAGGCACT-3′	5′-CCTCCGTGGGGATAGAACTT-3′	188
MMP-9	NM_004994.2	5′-AAAGCCTATTTCTGCCAGGAC-3′	5′-GTGGGGATTTACATGGCACT-3′	157
MMP-2	NM_004530.4	5′-CACTTTCCTGGGCAACAAAT-3′	5′-TGATGTCATCCTGGGACAGA-3′	257
Ang-1	NM_001146.5	5′-GAAGGGAACCGAGCCTATTC-3′	5′-GCTCTGTTTTCCTGCTGTCC-3′	108
Ang-2	NM_001147.3	5′-GGGAAGGGAATGAGGCTTAC-3′	5′-AAGTTGGAAGGACCACATGC-3′	232
PDGF-B	NM_033016.3	5′-TTGTGCGGAAGAAGCCAATC-3′	5′-CTCCTTCAGTGCCGTCTTGT-3′	239
FGF-2	NM_002006.5	5′-AGAGCGACCCTCACATCAAG-3′	5′-TCGTTTCAGTGCCACATACC-3′	224
VEGFR-2	NM_002253.2	5′-AGCGATGGCCTCTTCTGTAA-3′	5′-ACACGACTCCATGTTGGTCA-3′	172
eNOS	NM_000603.4	5′-ACCCTCACCGCTACAACATC-3′	5′-GCTCATTCTCCAGGTGCTTC-3′	198
VE-cadherin	NM_001795.3	5′-CCTACCAGCCCAAAGTGTGT-3′	5′-GACTTGGCATCCCATTGTCT-3′	249
CD31	NM_000442.5	5′-ATGATGCCCAGTTTGAGGTC-3′	5′-ACGTCTTCAGTGGGGTTGTC-3′	172
CD34	M81104.1	5′-CAATGAGGCCACAACAAACA-3′	5′-GTGACTGGACAGAAGAGTTT-3′	101
CD133	NM_001145847.2	5′-CAGTCTGACCAGCGTGAAAA-3′	5′-GGATTGATAGCCCTGTTGGA-3′	223
CXCL-12	NM_199168.4	5′-TCAGCCTGAGCTACAGATGC-3′	5′-CTTTAGCTTCGGGTCAATGC-3′	161
CXCR-4	NM_001348059.2	5′-GGTGGTCTATGTTGGCGTCT-3′	5′-TGGAGTGTGACAGCTTGGAG-3′	227
GAPDH	NM_002046.3	5′-ATCACTGCCACCCAGAAGAC-3′	5′-TTCTAGACGGCAGGTCAGGT-3′	210

**Table 2:** Angiogenic factors released by CGFs.

Angiogenic Factors ^1^	(ng/mL)
VEGF	1.1 ± 0.1
TGF-β1	18.3 ± 1.2
BMP-2	0.01 ± 0.003
MMP-2	136.6 ± 25.5
MMP-9	490.7 ± 59.7

^1^ Angiogenic factors were analyzed by ELISA in CGF-CM obtained by incubating CGFs in M199 medium for 14 days.

**Table 3:** Angiogenic factors released by CGF-derived cells.

Angiogenic Factors ^1^	(ng/mL)
VEGF	0.021 ± 0.007
TGF-β1	1.5 ± 0.4
MMP-2	16.7 ± 3.7
MMP-9	109.6 ± 13.8

^1^ Angiogenic factors were analyzed by ELISA in the supernatant of CGF-derived cells after 48 h of incubation.
